# Divergent Hepatic and Adipose Tissue Effects of Kupffer Cell Depletion in a Male Rat Model of Metabolic-Associated Steatohepatitis

**DOI:** 10.3390/biology14081058

**Published:** 2025-08-15

**Authors:** Morena Wiszniewski, Diego Mori, Silvia I. Sanchez Puch, Camila Martinez Calejman, Cora B. Cymeryng, Esteban M. Repetto

**Affiliations:** 1 Laboratorio de Endocrinología Molecular (LEM), Centro de Estudios Farmacológicos y Botánicos (CEFYBO), Consejo Nacional de Investigaciones Científicas y Técnicas (CONICET)—Universidad de Buenos Aires, Paraguay 2155, 5th Floor, Buenos Aires C1121ABG, Argentina; mwiszniewski@fmed.uba.ar (M.W.); mcalejman@fmed.uba.ar (C.M.C.); cymeryng@fmed.uba.ar (C.B.C.); 2 Cátedra de Bioquímica General y Bucal, Facultad de Odontología, Universidad de Buenos Aires, Buenos Aires C1121ABG, Argentina; 3 Instituto de Investigaciones en Microbiología y Parasitología Médica (IMPAM), Consejo Nacional de Investigaciones Científicas y Técnicas (CONICET)—Universidad de Buenos Aires, Paraguay 2155, 12th Floor, Buenos Aires C1121ABG, Argentina; 4 Departamento de Bioquímica Humana, Facultad de Medicina, Universidad de Buenos Aires, Buenos Aires C1121ABG, Argentina; 5 Laboratory of Metabolic Diseases and Aging, Institut Pasteur Montevideo, Montevideo 11400, Uruguay; 6 Facultad de Bromatología, Universidad Nacional de Entre Ríos, Presidente Perón 1154, Gualeguaychú E2822ABA, Argentina

**Keywords:** fatty liver, Kupffer cells, insulin resistance, rats, FGF21, adipose tissue, inflammation, diet

## Abstract

Kupffer cells (KCs) play a pivotal role in the progression of metabolic-associated steatohepatitis (MASH). This study evaluated the impact of short-term KC depletion induced by gadolinium chloride (GdCl_3_) in a rat model of MASH. The intervention with GdCl_3_ effectively reduced KC markers CD68 and Clec4f, together with pro-inflammatory cytokines (IL-1β, TNFα, NOS2), without affecting anti-inflammatory markers (IL-10, MRC1). Histologically, GdCl_3_ reduced hepatocyte ballooning and NAS despite persistent steatosis. KC depletion was associated with decreased oxidative stress markers (TBARS, 3-nitrotyrosine) and antioxidant enzyme activity (SOD, catalase). Additionally, markers of endoplasmic reticulum stress (ATF4, GRP78, CHOP, P58^IPK^) and apoptosis (BAX/BCL2 ratio, cleaved caspase-3) were diminished. Despite these improvements, GdCl_3_ had no effect on lipid or glucose metabolism in the liver, associated with persistent elevation of PTP1B expression induced by SRD intake. KC depletion, however, increased FGF21 expression. GdCl_3_ treatment improved systemic insulin sensitivity and reduced fasting glucose and NEFA serum levels. In white adipose tissue, the treatment decreased adipocyte size, restored insulin signaling, and inhibited lipolysis (ATGL expression) without altering macrophage infiltration (IBA) or thermogenic protein levels (UCP1) in SRD rats. These findings suggest that KC depletion modulates liver-to-adipose tissue crosstalk, potentially through FGF21 signaling, contributing to improved systemic metabolic homeostasis of SRD animals.

## Simple Summary

Metabolic-associated steatohepatitis (MASH) is a progressive form of steatotic liver disease characterized by steatosis, inflammation, and hepatocellular injury. Our study aimed to elucidate the hepatic and systemic effects of Kupffer cell depletion in a rat model of sucrose-induced MASH. This intervention, using gadolinium chloride, effectively depleted Kupffer cells, which correlated with a significant attenuation of hepatic inflammation, oxidative stress, endoplasmic reticulum stress, and apoptosis. Despite these improvements, hepatic steatosis, hepatic insulin resistance, and the expression of genes related to lipid metabolism and glucose output remained unaltered. Systemically, Kupffer cell depletion restored insulin sensitivity, evidenced by an improved insulin tolerance test, and enhanced white adipose tissue metabolism, including restored insulin signaling and reduced lipolysis, possibly mediated by the liver induction of fibroblast growth factor 21 associated with Kupffer cell depletion. We conclude that while Kupffer cells are pivotal mediators of inflammation and hepatocyte damage in MASH, the underlying hepatic metabolic dysregulation is sustained by cell-intrinsic mechanisms, likely involving fructose-induced protein tyrosine phosphatase type 1B expression. Our findings suggest that Kupffer cells can mediate inter-organ communication, and resolving MASH requires a dual therapeutic approach targeting both Kupffer cell-mediated inflammation and hepatocyte-intrinsic metabolic pathways.

## 1. Introduction

The liver serves as a metabolic hub, orchestrating the body’s response to nutrient intake and storage while integrating signals from both the adipose tissue and gastrointestinal tract. As the first organ to receive nutrient-rich blood via the portal vein, it regulates glucose, lipid, and amino acid metabolism in response to food intake [1]. Given its close relationship with the external compartment, the liver also represents the first line of defense against environmental changes and invading pathogens derived from the gut [2]; therefore, it is equipped with a great variety of immune cells.

Metabolic dysfunction-associated steatotic liver disease (MASLD), previously referred to as non-alcoholic fatty liver disease (NAFLD), comprises a variety of chronic liver disorders and is characterized by fat accumulation in at least 5% of hepatocytes, excluding other possible etiologies associated with fat accumulation [3]. It incorporates the metabolic factor in its name, since it is considered the hepatic manifestation of the metabolic syndrome [4]. The global prevalence of MASLD, currently at epidemic proportions, increases in parallel with global obesity rates [5], affecting 38% of the global population [6]. Metabolic-associated steatohepatitis (MASH) is a severe form of MASLD characterized by the presence of lobular inflammation and hepatocyte injury (ballooning) in addition to steatosis [7], and it significantly increases the chances of developing cirrhosis or hepatic carcinoma.

Although of unknown origin, it has been proposed that MASLD progression follows a model called “multiple parallel hits” [8]. This theory proposes that the establishment of steatosis sensitizes the liver to secondary hits (like oxidative stress, inflammation, the unfolded protein response, apoptosis), which would trigger disease progression [5].

Overnutrition caused by the lack of physical activity and the consumption of unhealthy diets is one of the main determinants in the progression of MASLD [6]. Although excess consumption of any food can lead to the development of MASLD, mono- and disaccharides, especially fructose and sucrose, can activate programs of hepatic de novo lipogenesis further exacerbating MASLD [9]. Recently it has been acknowledged that the consumption of sugar-sweetened beverages, due to their liquid form, leads to rapid consumption and digestion, resulting in lower satiety and higher caloric intake and weight gain [10] when compared to their solid counterparts. High doses of rapidly digested glucose and fructose can lead to systemic insulin resistance, adipose tissue dysfunction, ectopic fat deposition, and the establishment of metabolic dysfunction [10].

In the context of MASLD, Kupffer cells play a pivotal role in perpetuating the inflammatory response to metabolic stress. They register metabolic injury indirectly via damage-associated molecular patterns and directly via toxic metabolites, such as mitochondrial DNA released from hepatocytes or cholesterol, respectively [11]. Upon activation, Kupffer cells adopt a pro-inflammatory phenotype, secreting many different cytokines that promote the recruitment of monocyte-derived macrophages in the tissue, further amplifying hepatocyte metabolic injury in a positive feedback loop [12], thus promoting liver disease.

To maintain homeostasis and adapt to external cues, different tissues communicate with one another via multiple signals such as hepatokines, adipokines, or cytokines, ensuring appropriate metabolic flexibility [13]. White adipose tissue (WAT) is the major fat-storing depot and serves as the largest endocrine organ to secrete adipokines and cytokines systematically [14]. Adipokines are involved in various metabolic and physiological signaling cascades regulating glucose uptake, insulin signaling, fatty acid oxidation, and other energy-producing and metabolic processes in distant organs. In the context of obesity and insulin resistance, WAT plays a pivotal role in the metabolic systemic dysfunction characteristic of the disease. In obesity, WAT expands as the result of the energetic imbalance, leading to adipocyte dysfunction, chronic low-grade inflammation, and altered adipokine secretion, with the consequent modification of inter-organ communication, contributing to the spectrum of the disease. WAT influences liver metabolism not only through the synthesis of signaling hormones but also through the release of non-esterified fatty acids, the main resource for fat deposition in the liver [1]. In this context, the liver can also influence WAT metabolism and inflammation through the secretion of cytokines and hepatokines, indicating the presence of a vicious cycle promoting metabolic dysfunction.

Fibroblast growth factors are a family of hormones with described paracrine, autocrine, and endocrine functions [15]. Although FGF21 is expressed in a plethora of tissues (adipose, liver, pancreas, and muscle), circulating levels of this hormone are known to be derived primarily from the liver under physiological conditions in both rodents and humans [15]. Levels of FGF21 are increased under a variety of physical conditions including high carbohydrate diets [16], and its highest expression is achieved with the combination of high-carbohydrate and low-protein diets [16]. While FGF21 has been considered a stress hormone that helps the body cope with nutrient restriction, both circulating and tissue levels of this hormone are elevated in obesity, suggesting the presence of FGF21 resistance as an adaptive response [17].

It has been acknowledged that Kupffer cells can act as mediators of the function of distant organs, like WAT, through the release of cytokines [18,19]. Taking this into account, influencing liver inflammation by the inhibition of pro-inflammatory pathways or by stimulating anti-inflammatory pathways seems to be a promising therapeutic approach for MASH treatment [20]. However, information is still scarce regarding the specific role of Kupffer cells in the progression of the disease, and little is known about the effect of Kupffer cell ablation in the peripheral tissues’ metabolism.

Our group previously described and characterized a MASH model generated in male Wistar rats by the administration of 30% sucrose in the drinking water (SRD) for 12 weeks. Histologically, SRD induces steatosis and tissue injury, evidenced by the presence of ballooning without the development of fibrosis in the liver. SRD-fed animals presented systemic parameters of insulin resistance (moderate hyperglycemia and elevated serum triglyceride and NEFA levels). However, no changes in serum AST or ALT levels were observed. We detected liver inflammation, oxidative stress, activation of the unfolded protein response, apoptosis, and characteristic metabolic alterations of hepatic insulin resistance in these animals [21].

Taking this into account, the aim of this study is to assess the role of Kupffer cells in a MASH model and determine the impact of KC depletion in WAT and liver metabolism.

## 2. Material and Methods

### 2.1. Animal Model

Thirty-six male Wistar rats were acquired from the animal facility of Facultad de Farmacia y Bioquímica, Universidad de Buenos Aires. Animals were housed in groups of three and maintained under controlled conditions of humidity and temperature (21 ± 2 °C and 40–70% of RH) under a 12/12 h light–dark cycle for an adaptation period of one week. After this period, all animals began the experimental protocol, weighing 200–250 g. They were then randomly divided into those who received a regular chow diet and tap water (Control group) or sucrose (commercially available sugar, Ledesma group, Buenos Aires, Argentina) 30% *w*/*v* in the drinking water ad libitum for 10 weeks. After this time point, sucrose-fed animals were divided into two subgroups: those receiving 10 mg/kg gadolinium chloride in NaCl 0.9% i.p. (GdCl_3_, Sigma Aldritch, St. Louis, MO, USA) (SRD + Gd Group) and those receiving only vehicle (NaCl 0.9%, SRD Group) every three days until the end of the 12th week. Water and food consumption were monitored and recorded every 48 h, and animal weights were controlled weekly throughout the duration of the protocol.

The study design complied with the ARRIVE guidelines and was carried out in accordance with the Animal Care and Use Committee from Facultad de Ciencias Médicas, Universidad de Buenos Aires.

### 2.2. Tissue and Serum Collection

At the end of the experimental period, animals were fasted for 4 h and then sacrificed by decapitation in the morning. Livers and trunk blood were collected. After the obtention of serum, all samples were immediately frozen at −70 °C until further use.

### 2.3. Insulin Signaling Pathway

In a second set of experiments, at the end of the 12th week and 10 min before sacrifice, 12 animals (*n* = 4 per group) were injected with 1 IU/Kg insulin, i.p. (Sanofi, Paris, France). Epidydimal adipose tissue (eWAT) and livers were collected to assess insulin-stimulated AKT/PKB phosphorylation by Western blot.

### 2.4. Systemic Biochemical Parameters

Metabolic parameters such as glycemia, triglyceridemia, and cholesterolemia were determined using a Siemens ADVIA 1650 autoanalyzer, according to the manufacturer instructions (Siemens, Munich, Germany). Non-esterified fatty acid (NEFA) levels were measured using a colorimetric kit, according to the manufacturer specifications (RANDOX, Crumlin, UK).

### 2.5. Insulin and Pyruvate Tolerance Tests

For the insulin tolerance test (ITT), during the 12th week of treatment, animals were fasted for 4 h and were injected with 1 IU/kg of insulin i.p. Blood was drawn from the tip of the tail before and every 5 min after insulin injection up to 25 min. For the pyruvate tolerance test (PTT), animals were fasted for 6 h and then blood was drawn at 0, 15, 30, 60, 90, 120 min after the administration of 1 g/kg of sodium pyruvate (Sigma Aldritch, St. Louis, MO, USA, pH 7.4 diluted in sterile PBS 1×).

After both tests, serum was obtained and glucose levels were determined using a colorimetric method according to the manufacturer instructions (Wiener, Buenos Aires, Argentina). For the ITT, the slope of the curve of glucose concentration as a function of time (kITT) was calculated, while for the PTT, the area under the curve of glucose production was obtained.

### 2.6. Liver Triacylglyceride Content, Tissue Sectioning, and Staining

Hepatic TAG content was estimated as previously described [21]. For hematoxylin and eosin (H&E) staining, fresh liver fragments of 10 mm were fixed in 10% formaldehyde (pH 7.4) overnight at 4 °C. Formalin-fixed liver fragments were dehydrated, cleared in xylene, and embedded in paraffin. Serial longitudinal sections 5 µm thick were obtained with a microtome (Leica Microsystems, Buenos Aires, Argentina), and H&E staining was performed according to the manufacturer instructions (Biopack, Buenos Aires, Argentina). A qualified pathologist, blinded to the treatments, assessed these sections to determine the histopathological non-alcoholic fatty liver disease activity score (NAS) [22].

For immunofluorescence and Oil red O staining, freshly cut liver fragments were incubated overnight at 4 °C with 10% formal calcium fixative (1% calcium chloride in 10% formaldehyde). Samples were then incubated with increasing concentration of sucrose (15% to 30% g/mL) in 1× PBS, embedded in Tissue-tek OCT compound (Sakura Finetek, Tokyo, Japan), and sliced into 8 µm thick sections using a cryostat (Leica Microsystems, Buenos Aires, Argentina).

Immunofluorescence analysis was performed on cryostat sections, as described previously [21]. The antibodies used in this study were as follows: CLEC4f 1:20, AF2784, and 3-nitrotyrosine (1:50, MAB3248) (R&D systems, Minneapolis, MN, USA). TRICT donkey anti-goat IgG (1:100, 705-025-003) and AF488 goat anti-rabbit IgG (1:500, 711-545-152) (Jackson ImmunoResearch, West Grove, PA, USA). Images were acquired in an epi-fluorescence microscope (Zeiss Axio Vert A.1, Oberkochen, Germany) coupled to a digital camera (Axiocam MRc, Zeiss, Oberkochen, Germany.). Comparative digital images from different samples were obtained using identical exposure time settings. For image analysis, only settings of brightness and contrast were equally modified between treatments using Photoshop (Adobe Photoshop CS6, San Jose, CA, USA). Data is shown as median plus rank. ImageJ Software was used to quantify the fluorescence intensity (Version 1.53, NIH, Bethesda, MD, USA).

Oil red O staining was performed according to the manufacturer’s instructions (Biopack). All images were digitally captured using a light microscope (Eclipse E400, Nikon, Tokyo, Japan) equipped with a 6 V halogen lamp (20 W; equipped with a stabilized light source) and a camera (Coolpix s10, Nikon, Abingdon, VA, USA). To quantify Oil red O positive area, six random fields per slide were captured from six different liver samples per group at 200× magnification. The red pixel area was measured using ImageJ software. Data is presented as mean ± SEM of the percentage signal relative to control samples.

### 2.7. RNA Isolation and qPCR

RNA was isolated from 50 mg of hepatic tissue according to the manufacturer instructions (Quickzol^®^, Buenos Aires, Argentina). Retrotranscriptase reactions were performed in 2 µg total RNA using a MMLV (FCEN, Buenos Aires, Argentina). PCR reactions were performed on the obtained cDNA using the Bio-Rad CFX96 detection system (Bio-Rad, Hercules, CA, USA). Ct values were obtained using the corresponding software (CFX Maestro 1.1 version 4.1.2433.1219). Gene expression was normalized to the housekeeping gene β-Actin and quantified using the ΔΔct method corrected by the efficiency [23]. Primers and accession numbers used in this study are detailed in Table 1.

### 2.8. Protein Isolation and Western Blot

Liver tissue fragments were homogenized in RIPA buffer containing proteases and phosphatases inhibitor cocktails (Sigma Aldritch, St. Louis, MO, USA) for the isolation of proteins from whole-tissue homogenate. For the isolation of nuclear fractions, a differential centrifugation protocol was performed. Briefly, 100 mg of liver tissue was homogenized in 400 µL of homogenization buffer (50 mM phosphate buffer pH 7.4; 0.2 mM EDTA and 250 mM sucrose with a cocktail of protease inhibitors). Homogenates were then centrifuged at 2000× *g* for 10 min. Supernatants were transferred to a new tube and then centrifuged at 9000× *g* for 20 min. The pellet, corresponding to the nuclear fraction, was then resuspended in homogenization buffer.

Proteins were then quantified, loaded into an SDS-PAGE gel and transferred as previously described [21]. Once transferred, membranes were blocked with either 5% skim milk or 2% BSA in TTBS 1× for 1 h. Then, membranes were incubated with the specific primary antibody overnight at 4 °C: pAKT1^SER473^ 1:5000 (D4D6D), ATGL 1:1000 (#2138), ATF4 1:500 (D4B8) and cleaved caspase 3 1:500 (#9661) from Cell Signaling, Danvers, MA, USA. β-Actin 1:500 (C4 sc-47778), UCP1 1:5000 (sc-6528), TOM20 1:500 (sc-11415), GRP78 1:500 (sc-166490), and Lamin B (sc-6217) from Santa Cruz Biotechnology, Dallas, TX, USA. The following day, after several washes with TTBS 1×, the membranes were incubated with the appropriate secondary antibody at room temperature for 1 h, (either HRP-conjugated goat anti-rabbit (170-6515) from Bio-Rad, USA, or HRP-conjugated goat anti-mouse, (HAF007) from R&D Systems, USA. Specific chemiluminescence bands were detected using GeneGenome XRQ (Syngene, Bangalore, India). Intensity was quantified using the software FluorChem version 3.3 (Protein Simple, San Jose, CA, USA).

### 2.9. TBARS and Antioxidant Enzymes Activity

Lipoperoxide levels were estimated as thiobarbituric acid reactive substances (TBARS), as previously described [21]. For the determination of superoxide dismutase (SOD) and catalase activities, liver tissue was homogenized in 50 mM phosphate buffer (NaH_2_PO_4_/H_3_PO_4_), pH 7.40, and then centrifuged for 10 min at 3000 rpm at 4 °C. Samples were then diluted 1:10 with the same buffer. For catalase activity, a H_2_O_2_ solution was added, and its disappearance was monitored at 240 nm for 2 min. The activity is expressed as mM H_2_O_2_/min/µg. SOD activity was measured by its capacity to prevent the autooxidation of a solution of 4 mM pyrogallol (prepared in 10 mM HCl) in an alkaline medium (50 mM Tris–1 mM EDTA, pH 8.8), monitored at 420 nm for 2 min. Results are expressed as U/mg of protein.

### 2.10. Data Analysis

Data was assessed using the Shapiro–Wilk test for normal distribution. Normally distributed data are reported as mean ± SEM; otherwise, the median and rank are provided. Group differences were evaluated using one-way ANOVA or the Kruskal–Wallis test, depending on the distribution of data. Statistical analysis of data showing significant differences (*p* < 0.05) between groups were followed by post hoc Tukey’s or Dunn’s tests for group comparisons. All analyses were conducted using Prism 8 for Windows (GraphPad Software version 8.0.2, Solana Beach, CA, USA).

## 3. Results

### 3.1. Gadolinium Chloride Depletes the Kupffer Cell Population in the Liver

**Figure 1:**
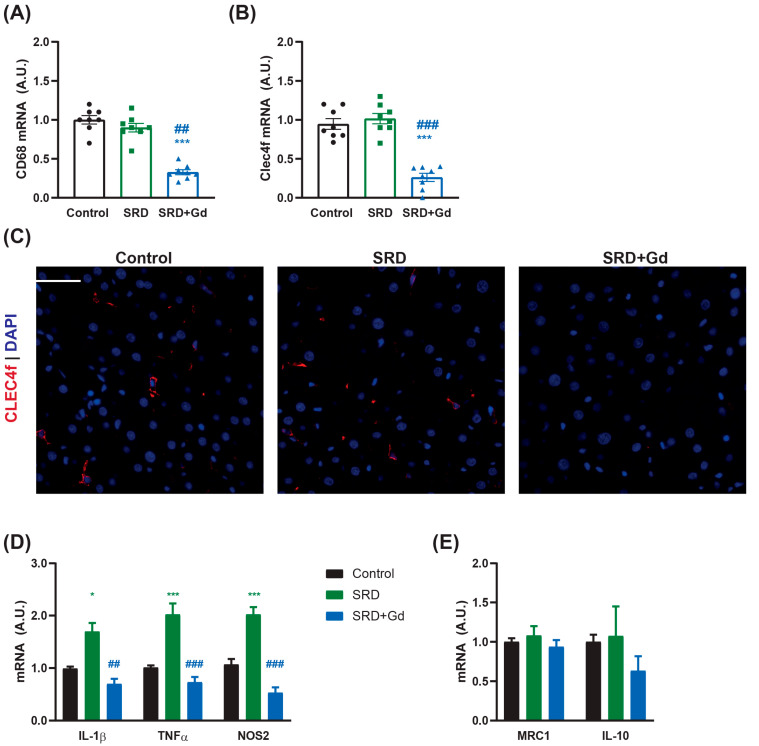
Gadolinium chloride treatment for the last two weeks of diet depletes Kupffer cells and reduces the production of pro-inflammatory cytokines by sucrose-rich diet (SRD). mRNA levels of (**A**) CD68 and (**B**) Clec4f assessed by qPCR. (**C**) Immunofluorescence staining was performed and observed at 400× magnification. Clec4f = red, DAPI = blue. White bar = 50 µm. mRNA levels of (**D**) pro-inflammatory markers and (**E**) anti-inflammatory markers assessed by qPCR. Data is shown as mean ± SEM of *n* = 8 per group. Statistical significance was obtained by one-way ANOVA followed by Tukey’s post hoc test; * *p* < 0.05, *** *p* < 0.001 vs. control; ^##^ *p* < 0.01 and ^###^ *p* < 0.001 vs. SRD group.

Our short-term treatment with gadolinium chloride (GdCl_3_) did not impact weight gain, food and liquid consumption, and caloric intake, as shown in Table 2. As expected, treatment with GdCl_3_ was associated with a significant reduction in the mRNA levels of CD68 and the specific Kupffer cell marker Clec4f, as shown in Figure 1A–C. This reduction correlated with a decreased expression of pro-inflammatory parameters, including IL-1β, TNFα, and NOS2, while the expression of anti-inflammatory markers, such as IL-10 and MRC1, remained unchanged (Figure 1D–E) when compared to the SRD or control groups.

### 3.2. Effect of Gadolinium Administration on Liver Histological Parameters

**Figure 2:**
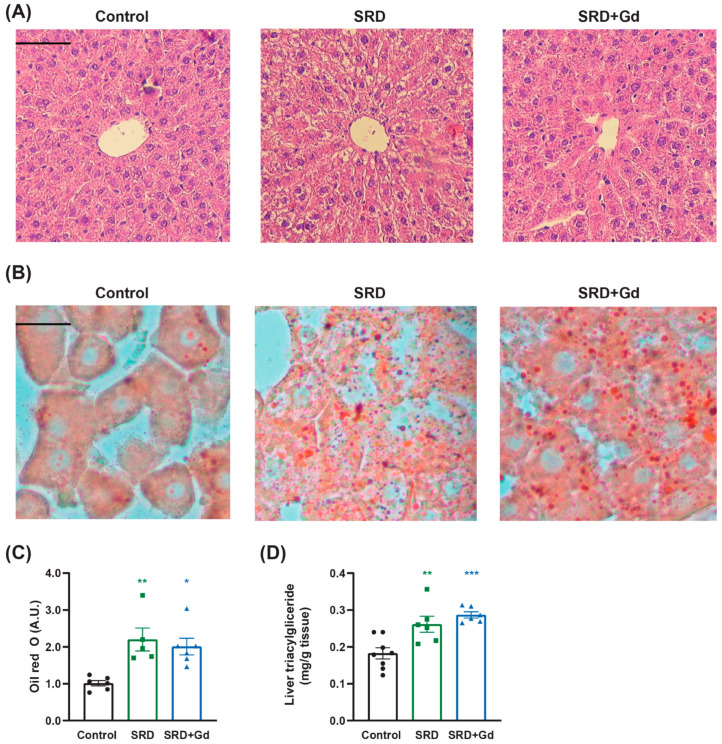
Liver histological effects of gadolinium chloride administration in a MASH model. Liver tissue sections 5 or 8 µm thick were stained with either (**A**) hematoxylin and eosin or (**B**) Oil red O staining. In both cases, images are shown at 200× magnification. A scale bar of 50 µm is provided. (**C**) Oil red O positive area. (**D**) Hepatic triglyceride content was measured. Data is shown as mean ± SEM of *n* = 7 per group. Statistical significance was obtained by one-way ANOVA followed by Tukey’s post hoc test; * *p* < 0.05, ** *p* < 0.01, *** *p* < 0.001 vs. control.

A two-week administration of GdCl_3_ to SRD-treated rats was enough to mitigate ballooned hepatocyte injury, as evidenced by H&E staining and by a reduction in the NAS (Figure 2A, Table 3). However, despite this intervention, SRD-associated steatosis persisted, as observed in Oil red O staining and its quantification, as well as in the hepatic triacylglyceride content (Figure 2B–D).

### 3.3. Kupffer Cell Depletion Is Associated with a Decrease in Oxidative Stress Markers

**Figure 3:**
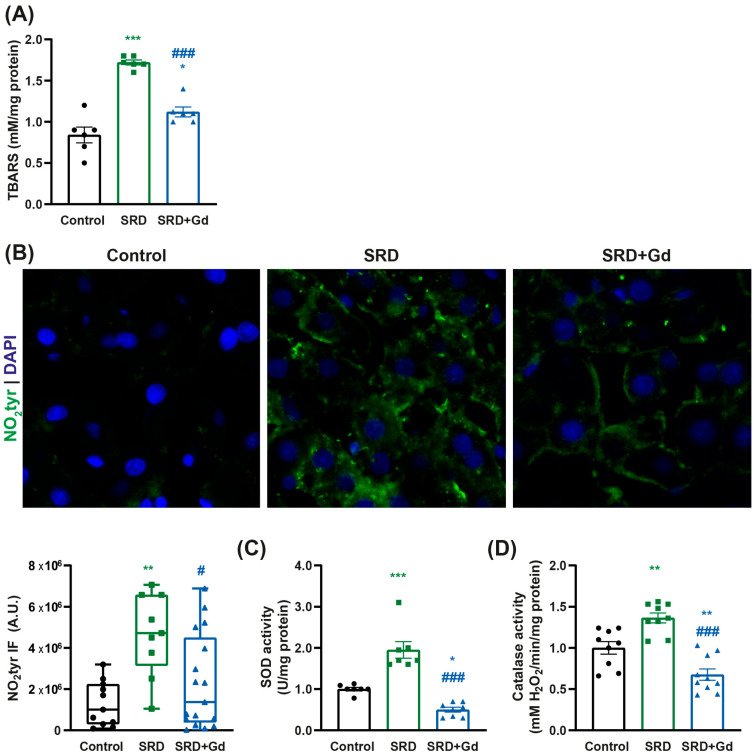
Kupffer cell depletion is associated with a reduction in oxidative stress markers. (**A**) Lipid peroxidation was evaluated by measuring thiobarbituric acid reactive substances (TBARSs). (**B**) Immunofluorescence staining and quantification were performed for proteins modified by 3-nitrotyrosine observed at 400× magnification. Data are displayed as median and the interquartile range, and statistical difference was obtained by Kruskal–Wallis followed by Dunn’s multiple comparison test; ** *p* < 0.01 vs. control; ^#^ *p* < 0.05 vs. SRD. (**C**) Superoxide dismutase (SOD) and (**D**) catalase activities were obtained from liver tissue homogenates. Data is shown as mean ± SEM of *n* = 8 per group. Unless stated otherwise, statistical significance was obtained by one-way ANOVA followed by Tukey’s post hoc test; * *p* < 0.05, ** *p* < 0.01, *** *p* < 0.001 vs. control; ^###^ *p* < 0.001 vs. SRD.

In SRD-GdCl_3_ rats we detected a reduction in oxidative stress markers, including lipoperoxides (as TBARS) and 3-nitrotyrosine-modified proteins (Figure 3A,B). Interestingly, this reduction was accompanied by a decrease in the activity of the antioxidant enzymes superoxide dismutase (SOD) and catalase in this group (vs. SRD), as shown in Figure 3C,D.

### 3.4. Kupffer Cell Depletion Is Associated with Reduced Cell Death

**Figure 4:**
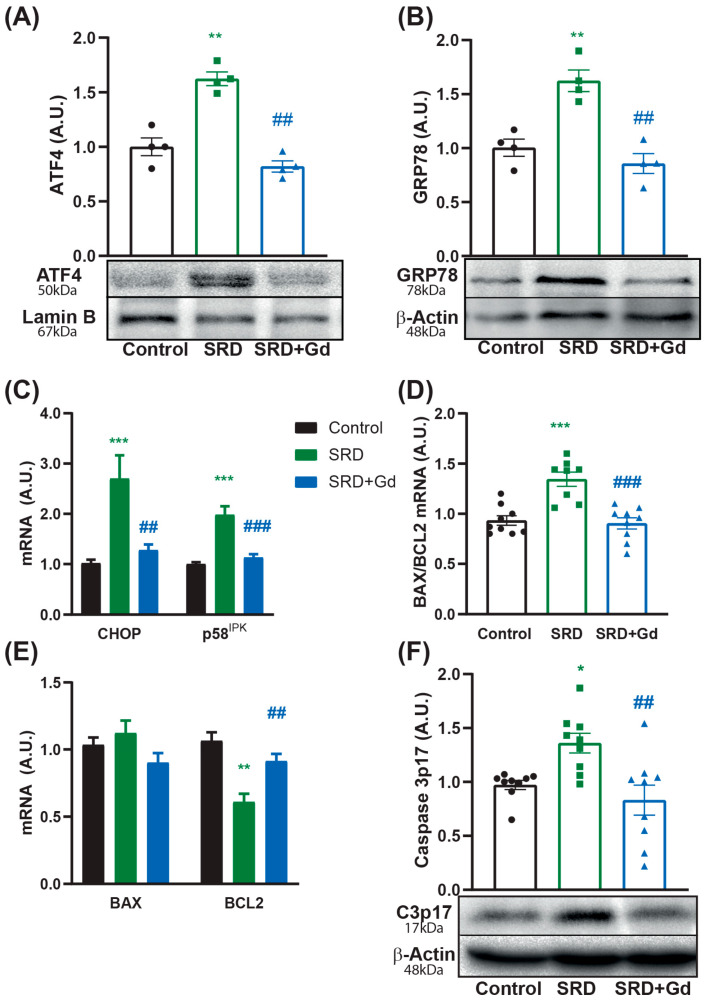
Endoplasmic reticulum stress and apoptosis markers are attenuated by Kupffer cell depletion. (**A**) Nuclear protein levels of ATF4 and (**B**) GRP78 protein levels were assessed through Western blot. (**C**) mRNA levels of unfolded protein response related genes, CHOP and P58^ipk^, were measured by qPCR. Apoptosis was measured through (**D**) mRNA levels by qPCR of the pro-apoptotic index BAX/BCL2. (**E**) BAX and BCL-2 levels are shown separately. (**F**) Protein levels of cleaved caspase 3 by Western blot (Supplementary Figure S1). Data is shown as mean ± SEM of *n* = 4–9 per group. Statistical significance was obtained by one-way ANOVA followed by Tukey’s post hoc test; * *p* < 0.05, ** *p* < 0. 01, *** *p* < 0.001 vs. control; ^##^ *p* < 0.01; ^###^ *p* < 0.001 vs. SRD group.

In our MASH model, Kupffer cell depletion was associated with a decrease in the expression of markers of the unfolded protein response, shown by a decreased nuclear translocation of ATF4, decreased protein levels of GRP78, and normalized mRNA levels of both CHOP and p58^IPK^, a pathway implicated in cellular damage associated with inflammation and oxidative stress (Figure 4A–C). These changes were accompanied by a reduction in the pro apoptotic index (BAX/BCL2 ratio) at the expense of the normalization of BCL2 mRNA levels (Figure 4D,E) and decreased protein expression of caspase 3 cleavage in the SRD + Gd group (Figure 4F), as compared with the SRD group.

### 3.5. Liver and WAT Metabolic Effects Associated with Kupffer Cell Depletion

**Figure 5:**
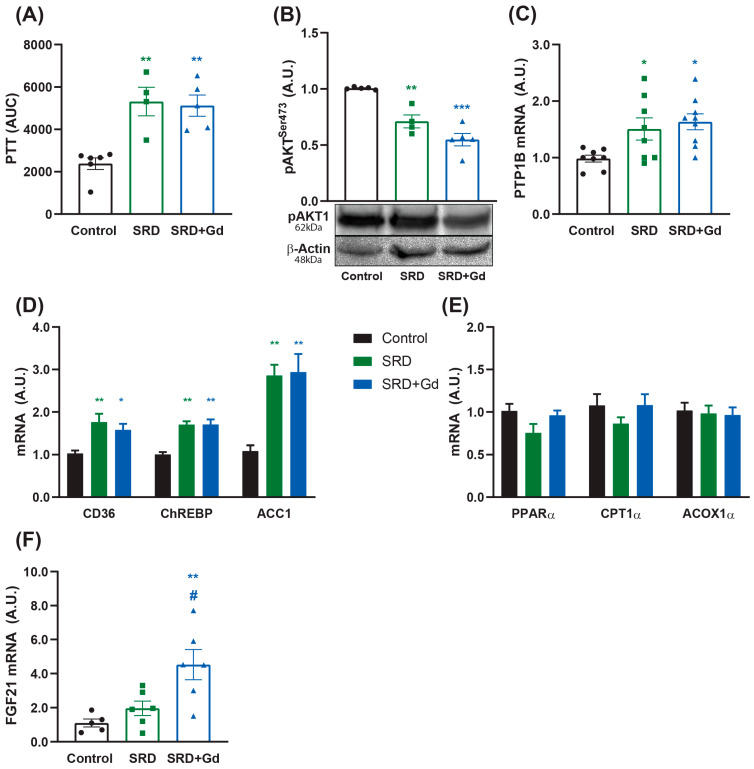
Liver metabolic effects associated with Kupffer cell depletion. (**A**) Pyruvate tolerance test was performed by the administration of 1 g/kg of sodium pyruvate in the 12th week of diet, after a 6 h fasting. (**B**) AKT phosphorylation levels were measured using Western Blot 10 min after the administration of 1 IU/kg of insulin to assess the hormone signaling in the liver. mRNA levels of (**C**) PTP1B, (**D**) lipogenic genes, (**E**) fatty acid oxidation related genes, and (**F**) FGF21 were measured by qPCR. Data is shown as mean ± SEM of *n* = 4–8 per group. * *p* < 0.05, ** *p* < 0.01, *** *p* < 0.001 vs. control. ^#^ *p* < 0.05 vs. SRD, assessed by One-way ANOVA followed by Tukey’s post hoc test.

GdCl_3_ treatment had no effect on hepatic glucose production during fasting, as assessed by the pyruvate tolerance test (Figure 5A). In addition, hepatic insulin signaling remained impaired in SRD-Gd treated rats, as evidenced by the phosphorylation levels of AKT, as well as the protein tyrosine phosphatase 1B (PTP1B) mRNA levels (Figure 5B,C). Kupffer cell depletion in SRD-treated rats did not modify the expression of genes involved in the lipid metabolism (Figure 5D,E). However, this treatment was associated with an increase in FGF21 levels when compared to both SRD and control groups (Figure 5F).

The insulin tolerance test, performed during the last week of treatment, indicated the restoration of systemic insulin sensitivity in the SRD + Gd group, as evidenced by an increased slope in the glucose disappearance curve when compared with the SRD group. Consistently, these animals presented significantly lower serum glucose and NEFA levels, without significant changes in either triglycerides or cholesterol levels when compared to the SRD group (Table 4).

**Figure 6:**
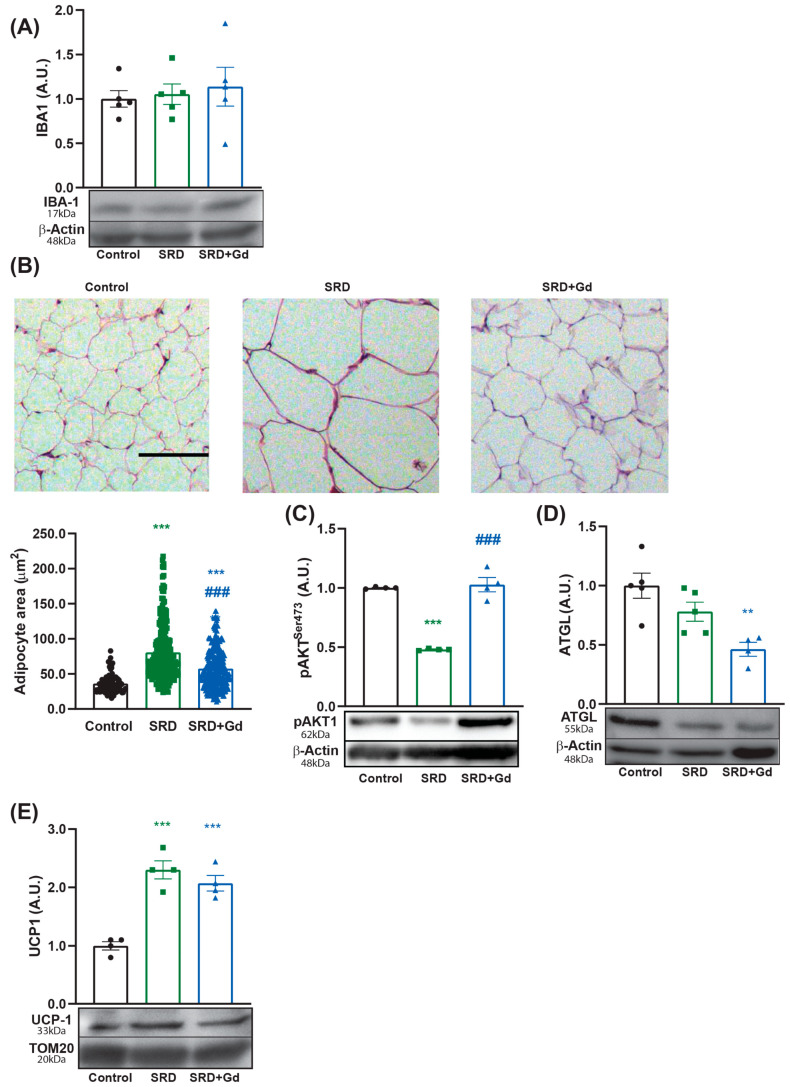
Metabolic impact of gadolinium chloride treatment in white adipose tissue (WAT) of SRD rats. (**A**) Protein levels of macrophage marker IBA-1 were assessed through Western Blot. (**B**) White adipose tissue sections of 5 µm thick were stained with hematoxylin and eosin; images are shown at 100× magnification. A scale bar of 100 µm is provided. (**C**) AKT phosphorylation levels were measured via Western Blot 10 min after the administration of 1 IU/kg of insulin to assess the hormone signaling. Protein levels of (**D**) ATGL and (**E**) UCP1 were measured by Western blot (Supplementary Figure S2). Data is shown as mean ± SEM of *n* = 4–8 per group. One-way ANOVA was followed by Tukey’s post hoc test; ** *p* < 0.01, *** *p* < 0.001 vs. control. ^###^ *p* < 0.001 vs. SRD.

In the epididymal white adipose tissue (WAT), GdCl_3_ treatment did not alter the levels of the macrophage marker IBA-1 (Figure 6A). However, it led to a significant reduction in adipocyte area (Figure 6B), a restoration of insulin-signaling, shown by increased pAKT levels (Figure 6C), and a reduction in a lipolysis enzyme levels (ATGL, Figure 6D). Protein levels of UCP1 remained elevated and unchanged compared to the SRD group (Figure 6E).

## 4. Discussion

In this study we examined the effects of Kupffer cells depletion by gadolinium chloride (GdCl_3_) in order to assess its therapeutic effect on MASH and its associated metabolic disturbances in rats fed a sucrose-rich diet. This contrasts with previous studies that focused on the early stages of MASLD through preventive depletion of Kupffer cells.

In the context of MASH progression, hepatic macrophages are thought to play a key role. In homeostatic conditions, yolk sac-derived Kupffer cells represent the main macrophage population in the liver. However, in MASLD, bone marrow-derived macrophages are recruited to the liver, contributing to different subsets of KCs while the main source of pro-inflammatory cytokines is thought to derive from monocyte-derived Kupffer cells (mo-KCs) [24,25]. Once having entered the liver tissue, this mo-KC population becomes indistinguishable from resident KCs, and the depletion approach to study this population does not distinguish between these subsets.

As expected, GdCl_3_ effectively depleted the Kupffer cell population in the liver, as confirmed by decreased mRNA expression of the pan-macrophage marker CD68, Clec4f, and its immunofluorescence analysis (Figure 1). Kupffer cell depletion was associated with marked reductions in the mRNA levels of pro-inflammatory cytokines, as detected in the SRD-group, including IL-1β, TNFα, and the pro-inflammatory phenotype marker NOS2 (M1 type macrophage), with no changes in M2-type phenotype markers IL-10 and MRC1. This is in agreement with the results presented by Bloomer SA et al., who described GdCl_3_ treatment, specifically reduced M1-type macrophages, without affecting M2-type macrophages in 24-week-old rats [26]. In addition, Pervin M et al. showed that M1-type macrophage markers were decreased in livers of rats pretreated with a dose of clodronate compared to controls after injection of a low dose of LPS, and although some M2 macrophage-related factors were increased (such as IL-4 or CSF-1), IL-10 was not one of them [27].

Histologically, GdCl_3_ treatment mitigated liver injury induced by the sucrose-rich diet, as evidenced by a reduction in hepatocellular ballooning, a hallmark of inflammatory injury in MASH patients [28]. These findings suggest that Kupffer cells are the main source of pro-inflammatory cytokines in our model and play a central role in MASLD progression, consistent with previous studies [12,29,30], and match the results of different studies that use gadolinium chloride to deplete Kupffer cells [31,32].

However, GdCl_3_ did not reduce the degree of steatosis compared to SRD treated rats (Figure 2 and Table 3). This could indicate that Kupffer cells primarily influence inflammation and injury rather than lipid accumulation. Steatosis could be the result of an alteration in hepatocyte metabolism or a consequence of excessive NEFA supply incorporated by the liver through their receptor CD36, as will be discussed below.

In the context of metabolic dysfunction, in the liver, oxidative stress could arise from multiple sources, including fructose metabolism [33], excessive fatty acid oxidation leading to mitochondrial dysfunction [5], and the macrophage-mediated respiratory burst in host defense mechanisms in response to the recognition of pathogen-associated molecular patterns or damage-associated molecular patterns [34].

Our results show that Kupffer cell depletion is associated with a decrease in the production of oxidative damage, assessed by a lower concentration of lipoperoxides as well as in proteins modified in 3-nitrotyrosine, accompanied by a decrease in the activity of antioxidant enzymes superoxide dismutase and catalase, as shown in Figure 3. This supports the notion that Kupffer cells are the main contributors to oxidative stress in the liver in our model of MASH, in agreement with others [35,36]. Similarly, after chronic alcohol exposure, Kupffer cells have been shown to promote oxidative stress by the expression of cytochrome P450 [37], and Kawai et al. demonstrated that the voltage-gated proton channels specifically expressed in Kupffer cells modulate the production of reactive oxygen species in the liver [38]. Furthermore, isolated Kupffer cells from MASH patients presented an increased staining of myeloperoxidase [39], and increased superoxide release when compared with cells from patients with simple steatosis.

The observed reductions in inflammation and oxidative stress—both key drivers of endoplasmic reticulum stress and the unfolded protein response [40,41], a molecular pathway associated with the progression of MASLD [42]—were accompanied by lower protein levels of ATF4 and GRP78, as well as reduced mRNA expression of CHOP and p58^IPK^, all markers of activation of this molecular pathway. This is also associated with reductions in the pro-apoptotic index (BAX/BCL-2), at the expense of a normalization of the mRNA levels of the anti-apoptotic protein BCL-2, and to the restored levels of caspase 3 cleavage, indicating reduced apoptosis and tissue injury (Figure 4). Apoptosis is a pivotal mechanism contributing to inflammation and fibrogenesis in MASH, and it can be activated by ER and oxidative stress, among other processes. Overexpression of CHOP by ER stress has been described as a plausible cause for BCL-2 decreased expression [43], and ROS production can induce caspase-3 cleavage [44]. Therefore, hepatocyte apoptosis has been acknowledged to be a generally prominent feature of MASH [45]. In this sense, the expression of anti-apoptotic BCL-2 has been shown to be diminished in both hepatocytes and serum, in accordance with the stage of MASLD/MASH. Additionally, TNFα secreted by activated Kupffer cells can induce apoptosis of adjacent hepatocytes, and the engulfment of apoptotic bodies by these cells further promotes the generation of this cytokine, thus creating a vicious cycle and triggering MASH progression [46]. As GdCl_3_ treatment attenuated both CHOP levels and TNFα production in our model, a reduction in pro-apoptotic markers was expected.

Despite a lower inflammatory grade and tissue oxidative stress, two molecular pathways associated with the development of hepatic insulin resistance [47,48], GdCl_3_ treatment did not restore hepatic insulin sensitivity or alter fasting hepatic glucose production, as evidenced by unaltered AKT phosphorylation and pyruvate tolerance tests, respectively (Figure 5). These results explain the persistence of glycemic levels above those of the control group, as seen in Table 4 [49]. In agreement, previous studies by Lanthier et al. demonstrated that Kupffer cell depletion with clodronate prevents metabolic alterations when performed concurrently with a high-fat diet but fails to reverse them once the metabolic disturbances are established [50]. Similar results were reported by Huang et al. [51].

Fructose intake is strongly associated with MASLD progression [52], and it has been identified as an independent risk factor for the development of hepatic insulin resistance [53]. Protein tyrosine phosphatase 1B (PTP1B) is a soluble enzyme that plays an essential role in the regulation of metabolism [54], specifically in the modulation of leptin and insulin sensitivity, marking it as an interesting therapeutic target for the treatment of type 2 diabetes and obesity. PTP1B is induced in the liver after fructose consumption, and its main function is to dephosphorylate tyrosine residues present in its substrates in the plasma membrane (for example, the insulin receptor and its substrates), negatively regulating the insulin signaling transduction pathway [53]. In the liver, PTP1B is also involved in the regulation of lipogenesis, as hepatocyte-specific PTP1B deficient mice presented improved glucose and lipid homeostasis in a high-fat feeding protocol [55]. Our results indicate that GdCl_3_ treatment did not affect the expression levels of PTP1B induced by SRD treatment, nor did it affect hepatic lipid metabolism, as evidenced by the lack of effect of GdCl_3_ treatment on the levels of enzymes involved in lipid synthesis and oxidation (Figure 5), systemic triglyceride levels (Table 4), and hepatic lipid content (Figure 2). These results suggest that the metabolic effects of fructose, in rats fed an SRD, are independent of Kupffer cell activity.

Insulin resistance is defined as a reduced response of sensitive tissues to insulin stimulation, and it can be influenced by both genetic and environmental factors [56]. Chronic inflammation, resulting from excessive nutrient intake, is the main environmental factor involved in the development of systemic insulin resistance, as it has been widely accepted that obesity, metabolic syndrome, and type 2 diabetes are characterized by the presence of chronic low-grade inflammation [57]. The recruitment, accumulation, and activation of pro-inflammatory macrophages in white adipose tissue, liver, and pancreatic islets are the ultimate driver of this chronic low-grade inflammation [57,58,59]. In particular, a crosstalk between tissue-resident macrophages and adipocytes or hepatocytes is involved in MASLD progression [60].

Our results show that the concomitant administration of GdCl_3_ with a sucrose-rich diet during the final two weeks of the dietary modification had no effect on weight gain, food/sucrose consumption, or caloric intake. However, this treatment restored systemic insulin sensitivity, characterized with a significantly reduced glycemia, a normalized response in an insulin tolerance test, and lower NEFA levels as compared to the SRD group (Table 2 and Table 4). Given the main role of white adipose tissue (WAT) in the regulation of systemic insulin resistance, these results could be explained by the effect of Kupffer cell depletion on WAT, as will be discussed below. These findings are consistent with those from Neyrinck et al., who showed that Kupffer cell inhibition abolished high-fat diet-induced hyperglycemia and glucose intolerance when administered alongside dietary modifications [61].

The main effects attributed to FGF21 are mediated through its interaction with the KLB receptor, expressed at high levels in the adipose tissue, acting as a ‘master sensitizer’ of specific hormonal signals, mainly insulin. Our results indicate that SRD feeding does not change the expression of FGF21 in the liver, yet its expression was increased in the group receiving gadolinium chloride (Figure 5). In agreement, Weide et al. showed that Kupffer cell depletion caused a reduction in the expression of the pro-inflammatory cytokine IL-1β associated with higher levels of FGF21 in hepatocytes [62].

In adipose tissue, FGF21 enhances insulin sensitivity, promoting glucose uptake and inhibiting lipolysis, as well as promoting UCP1 expression [15,63]. In agreement, our results indicate that animals in the SRD + Gd group present a reduction in serum levels of NEFA, and restored insulin signaling and reduced lipolysis in WAT. These effects did not correlate with changes in protein levels of the marker of macrophage content IBA-1, in agreement with Zeng et al., who demonstrated that intraperitoneal administration of GdCl_3_ twice weekly for 12 weeks did not alter the macrophage content of adipose tissue, as assessed by F4/80 and CD68 markers [32] (Table 4, Figure 6). Nevertheless, these findings do not exclude a potential direct effect of GdCl_3_ on the phenotype of WAT macrophages, which could influence insulin signaling both locally and systemically, as previously described. Our experimental approach does not allow us to assess if depletion of Kupffer cells directly contributes to WAT metabolism or if changes in WAT are secondary to hepatic alterations. However, we have shown that Kupffer cell depletion is associated with a reduction in pro-inflammatory markers, restauration of oxidative stress, the unfolded protein response, and apoptosis in the liver. More studies are needed to address this question.

While FGF21 is known to promote UCP1 expression in WAT, we observed that UCP1 levels were already increased by the sucrose-rich diet and were not further altered by GdCl_3_ treatment (Figure 6). This suggests that, in our model, chronic high-sucrose feeding is the dominant driver of UCP-1 induction, an effect that is independent of both Kupffer cell activity and the additional increase in FGF21. It has been suggested that an increased expression of UCP1 in WAT is a metabolic strategy to mitigate the effects of diet-induced obesity, promoting energy expenditure [13].

Our study has some limitations, such as the impossibility of determining FGF21 protein levels (neither in the liver nor in plasma) and the absence of functional assays to evaluate Kupffer cell activity. In addition, the use of more sophisticated techniques (such as single-cell RNA sequencing [64]) would allow for the proper classification and differentiation of resident macrophages in the liver in our MASH model.

## 5. Conclusions

Our study highlights the dual and divergent role of Kupffer cells in MASH progression. While they are critical drivers of hepatic inflammation, oxidative stress, and hepatocellular injury, their depletion alone is insufficient to reverse established hepatic insulin resistance and steatosis in rats fed a high-sucrose diet. We propose that Kupffer cell-independent mechanisms, such as SRD-induced PTP1B expression, sustain the hepatic metabolic dysfunction. Kupffer cell depletion, however, was associated with a marked improvement in WAT metabolic parameters, evidenced by restored systemic insulin sensitivity and reduced serum NEFA levels. We suggest that this liver–adipose tissue crosstalk is mediated, at least in part, by an increase in hepatokines such as FGF21, whose levels were increased following Kupffer cell depletion.

These findings strongly suggest that while targeting Kupffer cells is a valid anti-inflammatory strategy for the liver, its primary metabolic benefits may be exerted on peripheral tissues, highlighting the relevance of inter-organ crosstalk. A complete resolution of MASH will likely require combination therapies that not only tackle inflammation but also target metabolic pathways in both the liver and adipose tissue.

## Figures and Tables

**Table 1:** qPCR primers used in this study.

Target	Forward	Reverse	Accession Number
ACC1	ATGAACACCCAGAGCATTGTCCAG	AGCAGATCCATCACCACAGCCTT	NM_022193.2
ACOX1α	TCGAAGCCAGCGTTATGAGG	TTGAGGCCAACAGGTTCCAC	NM_001414015.1
BAX	CCAGGACGCATCCACCAAGA	AGCTGCCACACGGAAGAAGA	NM_017059.2
BCL2	GGCTACGAGTGGGATACTGGAGAT	CTCTCAGGCTGGAAGGAGAAGATG	NM_016993.2
CD36	CACAGGCTTTCCTTCTTTGC	CTCTGACATTTGCAGGTCCA	NM_031561.2
CD68	GCAATTCACCTGGACCTGCT	AGAGAGATTGGTCACTGGCG	NM_001031638.1
CHOP	CTCTGCCTTTCGCCTTTGA	GCTTTGGGAGGTGCTTGTG	NM_030186.1
ChREBP	ATAGAGGAGCTCAATGCTGCCAT	AACATGTCCCGCATCTGGTC	NM_001393706.1
CLEC4f	TGTCATCCTACAGCCCCAAG	GAGAGAGAAGAACACAGTCACC	NM_053753.1
CPT1α	ACGAAGCCCTCAAACAGA	GGATGAAATCACACCCAC	NM_031559.2
IL-10	GTTGCCAAGCCTTGTCAGAAA	TTTCTGGGCCATGGTTCTCT	NM_012854.2
IL-1β	CACCTCTCAAGCAGAAGCACAG	GGGTTCCATGGTGAAGTCAAC	NM_031512.2
MRC1	CAAGGAAGGTTGGCATTTGT	GGAACGTGTGCTCTGAGTT	NM_001106123.2
NOS2	CTTGGAGCGAGTTGTGGATTG	GGTGGGAGGGGTAGTGATGTC	NM_001429940
P58^IPK^	ATTAAAGCATACCGAAAGTTAGCAC	AGAGGGTCTTCTCCGTCATCAAA	NM_022232.2
PPARα	AAGAACCTGAGGAAGCCA	AGCCACAAAAAGGGAAATG	NM_013196.2
PTP1B	ACACAGTACGGCAGTTGGAG	GACTCCAAAGTCAGGCCAGG	NM_012637.2
TNFα	TGACCCCCATTACTCTGACC	GCAATCCAGGCCACTACTTC	NM_1278601
β-Actin	CCACACCCGCCACCAGTTC	GACCCATTCCCACCATCACACC	NM_031144

**Table 2:** Systemic metabolic characteristics. Data is shown as mean ± SEM. SRD: sucrose-rich diet; Gd: gadolinium chloride. ANOVA with Tukey’s post hoc test was performed according to data distribution; *** *p* < 0.001 vs. control group.

	Control	SRD	SRD + Gd
*n*	12	12	12
Initial weight (g)	205 ± 5	210 ± 7	204 ± 4
Final weight (g)	523 ± 9	639 ± 15 ***	612 ± 11 ***
Weight gain (g)	319 ± 9	429 ± 14 ***	411 ± 9 ***
Food consumption (g/rat/day)	31 ± 2	20 ± 1 ***	17 ± 1 ***
Water/sucrose consumption (mL/rat/day)	68 ± 2	69 ± 3	67 ± 2
Total kcal consumed	7490 ± 360	10,691 ± 467 ***	10,541 ± 419 ***

**Table 3:** Histopathological damage score. NAS (non-alcoholic fatty liver disease activity score) was performed in liver slides 5 µm thick, stained with hematoxylin and eosin. Data is shown as median and rank; ** *p* < 0.01, *** *p* < 0.001 vs. control; ^##^ *p* < 0.01; ^###^ *p* < 0.001 vs. SRD; assessed by non-parametric ANOVA (Kruskal–Wallis) followed by Dunn’s test.

	Control	SRD	SRD + Gd
*n*	8	8	8
Ballooning	0 (0–0)	2 (1–2) ***	1 (0–2) ***^###^
Steatosis	0 (0–0)	2 (1–3) ***	2 (1–3) ***
Lobular infiltration	1 (0–1)	1 (0–1)	0 (0–1)
Fibrosis	0 (0–0)	0 (0–0)	0 (0–0)
NAS	1 (0–1)	4 (5–3) ***	3 (0–5) **^##^

**Table 4:** Systemic metabolic effects of gadolinium chloride administration. After 12 weeks of diet, animals were sacrificed after 4 h fasting. Truncal blood was collected and serum separated. The specified determinations were performed. NEFA: non-esterified fatty acids. Data is shown as mean ± SEM; *** *p* < 0.001 vs. control; ^#^ *p* < 0.05; ^##^ *p* < 0.01 vs. SRD, assessed by one-way ANOVA followed by Tukey’s post hoc test.

	Control	SRD	SRD + Gd
*n*	12	12	12
kITT (mmol/L/min)	6.50 ± 0.50	3.63 ± 0.17 ***	5.77 ± 0.41 ^##^
Glycemia (mmol/L)	6.77 ± 0.23	8.45 ± 0.23 ***	7.41 ± 0.25 ^##^
Triacylglycerides (mmol/L)	1.00 ± 0.08	2.76 ± 0.25 ***	2.66 ± 0.18 ***
Total cholesterol (mmol/L)	1.88 ± 0.08	1.98 ± 0.09	1.98 ± 0.06
NEFA (mmol/L)	0.32 ± 0.02	0.67 ± 0.06 ***	0.47 ± 0.04 ^#^

## Data Availability

Full uncropped photos and Western blot bands are available in the following FigShare repository: https://doi.org/10.6084/m9.figshare.29433719.v1.

## Supplementary Materials

The following supporting information can be downloaded at: https://www.mdpi.com/article/10.3390/biology14081058/s1, Figures S1 and S2: uncropped Western blot bands.
