# Supplementary material for: Divergent Hepatic and Adipose Tissue Effects of Kupffer Cell Depletion in a Male Rat Model of Metabolic-Associated Steatohepatitis
Source: Biology (Basel). 2025 Aug 15;14(8):1058. doi: 10.3390/biology14081058 (PMC12383587; doi:10.3390/biology14081058)

ATF4 and Lamin B molecular weight and full uncropped Western blot

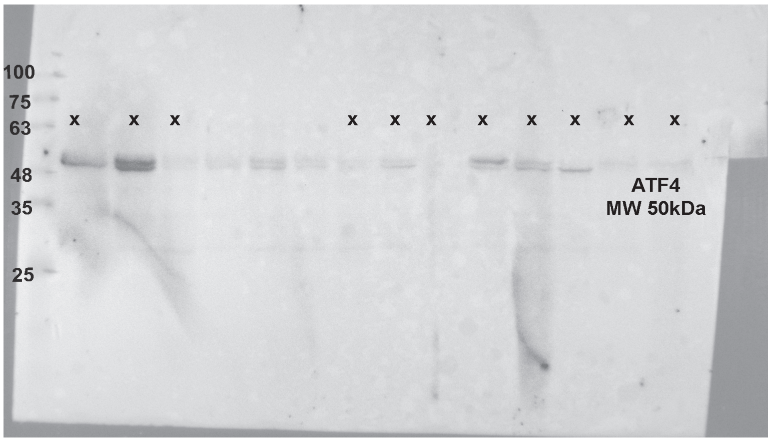

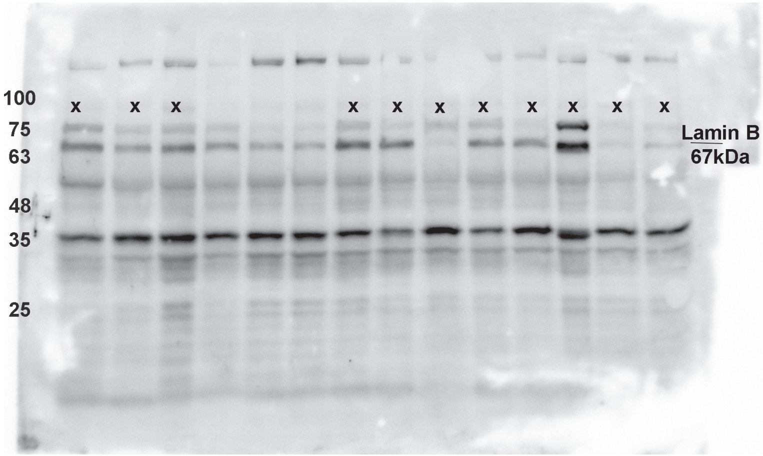

GRP78 and B-Actin molecular weight and full uncropped Western blot

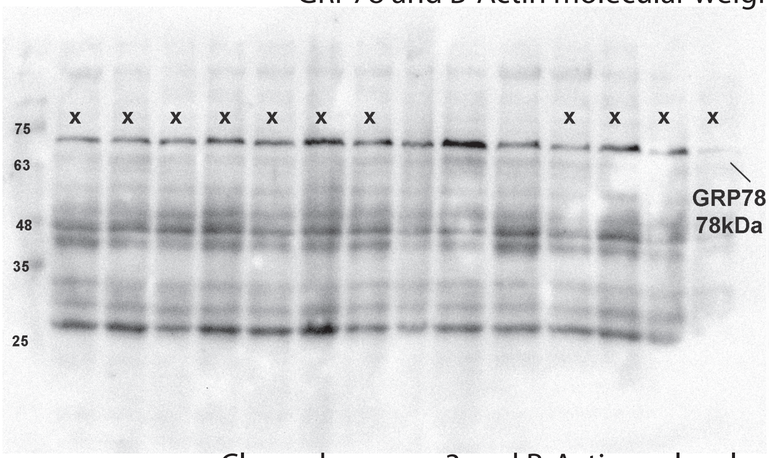

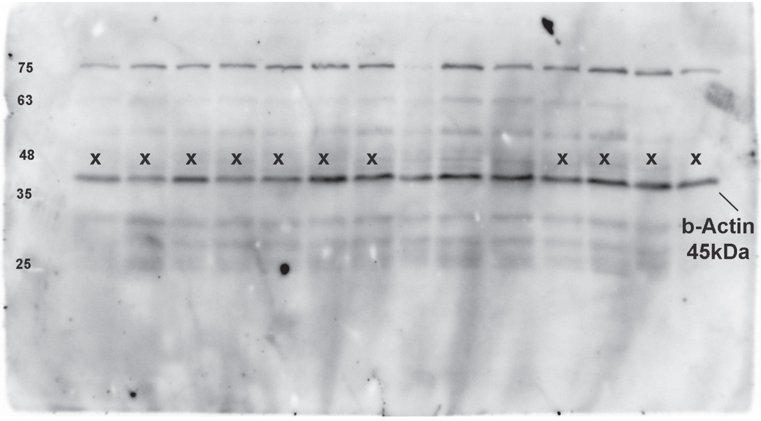

Cleaved caspase 3 and B-Actin molecular weight and full uncropped Western blot

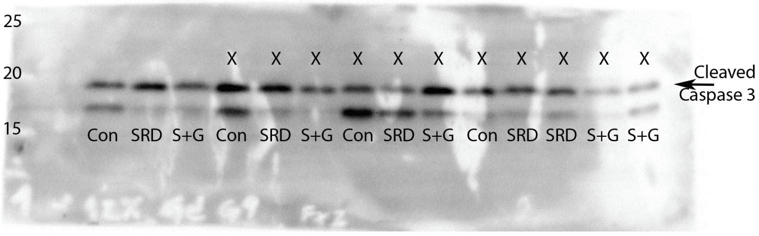

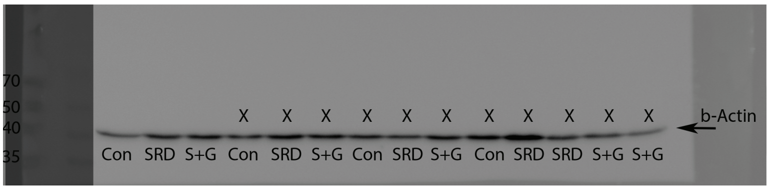

pAKT1 and B-Actin molecular weight and full uncropped Western blot

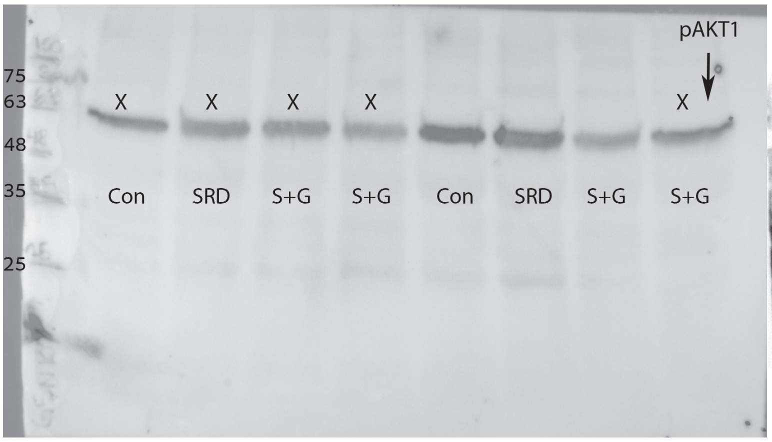

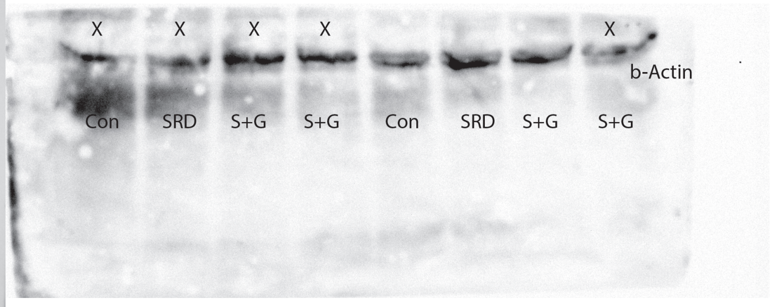

Supplement: Supplementary file 1 [file biology-14-01058-s001.zip › Supplementary Figure S1.pdf]
